# A Clustering Approach for Atmospheric Phase Error Correction in Ground-Based SAR Using Spatial Autocorrelation

**DOI:** 10.3390/s24134240

**Published:** 2024-06-29

**Authors:** Yaolong Qi, Jiaxin Hui, Ting Hou, Pingping Huang, Weixian Tan, Wei Xu

**Affiliations:** 1 College of Information Engineering, Inner Mongolia University of Technology, Hohhot 010051, China; qiyaolong@imut.edu.cn (Y.Q.); h2353854694@163.com (J.H.); hwangpp@imut.edu.cn (P.H.); wxtan@imut.edu.cn (W.T.); xuwei1983@imut.edu.cn (W.X.); 2 Inner Mongolia Key Laboratory of Radar Technology and Application, Hohhot 010051, China

**Keywords:** ground-based synthetic aperture radar (GB-SAR), atmospheric phase (AP), permanent scatterer (PS), spatial autocorrelation, complicated atmospheric condition

## Abstract

When using ground-based synthetic aperture radar (GB-SAR) for monitoring open-pit mines, dynamic atmospheric conditions can interfere with the propagation speed of electromagnetic waves, resulting in atmospheric phase errors. These errors are particularly complex in rapidly changing weather conditions or steep terrain, significantly impacting monitoring accuracy. In such scenarios, traditional regression model-based atmospheric phase correction (APC) methods often become unsuitable. To address this issue, this paper proposes a clustering method based on the spatial autocorrelation function. First, the interferogram is uniformly divided into multiple blocks, and the phase consistency of each block is evaluated using the spatial autocorrelation function. Then, a region growing algorithm is employed to classify each block according to its phase pattern, followed by merging adjacent blocks based on statistical data. To verify the feasibility of the proposed method, both the traditional regression model-based method and the proposed method were applied to deformation monitoring of an open-pit mine in Northwest China. The experimental results show that for complex atmospheric phase scenarios, the proposed method significantly outperformed traditional methods, demonstrating its superiority.

## 1. Introduction

Landslides, as highly destructive natural disasters, often pose severe threats to human life and property safety. Accurate landslide monitoring technology has become especially important to more effectively mitigate the losses caused by landslides [1]. Ground-based synthetic aperture radar (GB-SAR) technology, with its high precision and short revisit period, has shown significant advantages in landslide monitoring. Compared with traditional monitoring technologies, GB-SAR offers all-weather capabilities, wide spatial coverage, and fast image acquisition, making it more accurate in monitoring minute-by-minute or hourly changes in deformation areas [2]. This technology has been successfully applied in various fields, such as open-pit mine slope stability monitoring [3], dam safety monitoring [4,5], landslide rescue [6], and glacier monitoring [7].

When using GB-SAR technology for landslide monitoring, changes in atmospheric parameters such as temperature, humidity, and pressure cause temporal and spatial variations in the atmospheric refractive index, which affects the propagation speed and path of electromagnetic waves. This results in atmospheric phase errors in radar echoes. Severe atmospheric effects can significantly impact deformation inversion results, leading to misinterpretation of deformation areas [8]. Therefore, to effectively eliminate atmospheric phase errors, many scholars, both domestically and internationally, have conducted extensive research, which can generally be categorized into three types. The first type is the meteorological data correction method. This method assumes uniform atmospheric changes and quantitatively estimates atmospheric phase errors by introducing an atmospheric refractive index model combined with meteorological data (temperature, humidity, and atmospheric pressure) [9,10]. However, its accuracy highly depends on the location of meteorological stations and the acquisition and accuracy of meteorological data. Especially for distant targets in large research areas, observational errors in meteorological data can lead to significant correction errors. The second type is the model-based correction method. This approach establishes regression models related to distance, height, or angle and uses artificially set stable points [11] or permanent scatterers (PS) [12] to solve the regression coefficients, thereby estimating the atmospheric phase. The advantage of this method is that it does not require prior meteorological data, making it relatively simple and convenient to operate. It is currently the most widely used method. However, when monitoring targets in complex environments such as high altitudes or steep terrains, the drastic changes in atmospheric conditions directly affect the accuracy of atmospheric phase correction [13]. The aforementioned two methods, based on the assumption of atmospheric homogeneity, often fail to accurately estimate atmospheric phase components in such environments. To overcome this issue, a third type of method has been proposed in recent years: data-driven atmospheric phase correction techniques. These methods typically divide the atmospheric phase screen into multiple sub-blocks and correct these sub-blocks using interpolation or parameter estimation techniques to achieve high-precision atmospheric phase estimation [14,15,16,17]. These methods demonstrate higher flexibility and adaptability when dealing with complex atmospheric conditions, providing new and effective approaches for atmospheric phase correction.

To address the issue of atmospheric phase removal under complex atmospheric conditions, this paper proposes a spatial autocorrelation clustering-based atmospheric removal method. This method first uniformly partitions the extracted PS points into sub-blocks and then calculates the spatial autocorrelation of each sub-block. By setting a threshold, each sub-block is further subdivided into multiple categories based on phase variation trends. Subsequently, merging is performed based on the statistical characteristics of each category, and multivariate regression fitting is conducted for each merged category. Finally, interpolation is used to obtain a complete estimated atmospheric phase screen. This method allows for a more detailed capture of atmospheric phase error characteristics by analyzing local regions of the interferogram through spatial autocorrelation. To validate the practical application of this method, data collection and processing were conducted at an open-pit mine in Northwest China using a GB-InSAR system. By comparing the proposed method with traditional methods, the results demonstrated the superiority of the proposed method in correcting atmospheric phase errors under complex atmospheric conditions.

The rest of this article is organized as follows: Section 2 introduces related work, including the study area, the radar used, and the permanent scatterer selection method. Section 3 presents the ground-based radar interferometric signal model, processes the selected interferograms using traditional methods, and then introduces the clustering method based on spatial autocorrelation. Section 4 details the deformation monitoring experiment conducted at the open-pit mine and validates the proposed method’s effectiveness by comparing it with traditional methods. The discussion is presented in Section 5, and the conclusions are presented in Section 6.

## 2. Related Work

### 2.1. Study Area

**Figure 1:**
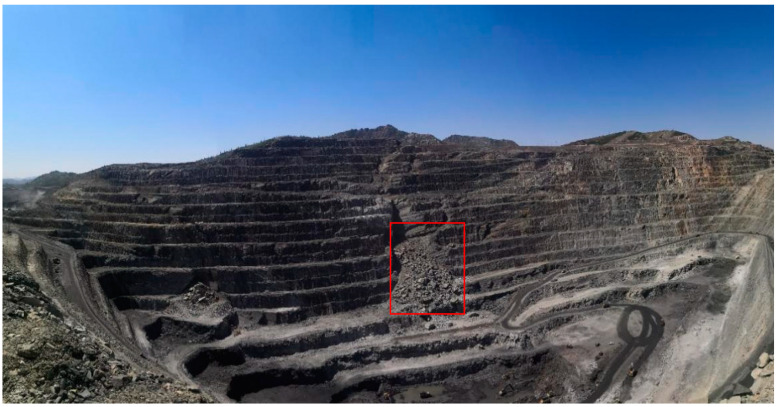
Photograph of the open-pit mine.

The experiment was conducted in an open-pit mine in northwestern China, spanning from 0:00 on 18 April 2021 to 10:00 on 21 April 2021. The mine is located at an altitude ranging from 1000 m to 1300 m, with notable topographical features where the northern part is higher than the eastern part, creating a relative height difference of 50 to 300 m. The geological structure of the mine is primarily composed of rock. A total of 225 SAR images were acquired during the experiment. An on-site optical image is shown in Figure 1. The radar was positioned facing the slope, covering the entire mine. The red box in the figure marks the location of the landslide that occurred during the observation period.

**Figure 2:**
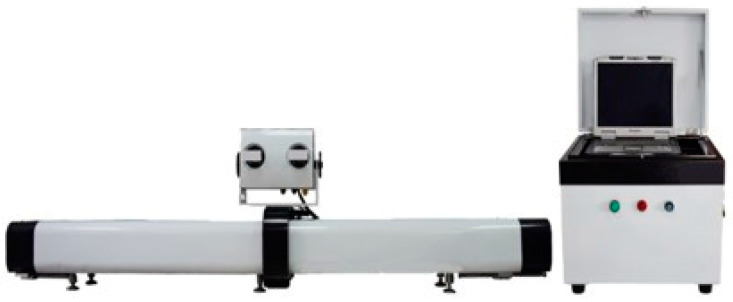
GB-SAR system.

The instrument used for observation was a linear scanning micro-deformation monitoring radar, which is a Ku-band GB-SAR system. A picture of the radar is shown in Figure 2, and the system parameters are listed in Table 1.

### 2.2. Permanent Scatterer Selection

**Figure 3:**
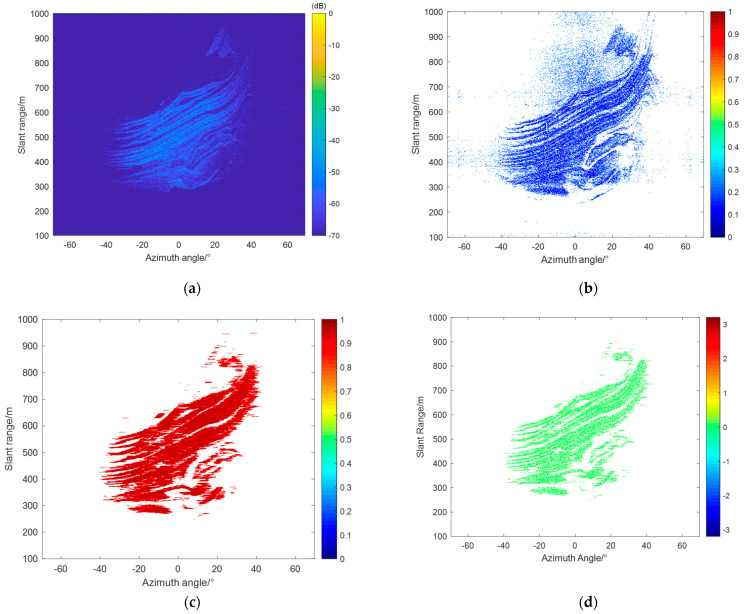
(**a**) Power image of the monitoring area. (**b**) Amplitude deviation index map. (**c**) Coherence coefficient map. (**d**) Permanent scatterer (PS) selection results, where the amplitude deviation index threshold was 0.25 and the coherence coefficient threshold was 0.9.

Figure 3a shows the radar image of the study area. To facilitate the study, this paper employs a dual-threshold method based on amplitude deviation [18] and coherence coefficient [19] to select permanent scatterers (PS). The scattering characteristics of PS points remain highly stable during long-term monitoring, with their phase typically influenced by atmospheric effects. PS points can thus be used to estimate the atmospheric phase screen. The amplitude deviation method identifies PS points based on the statistical properties of the amplitude of target points in the radar complex image over a time series, typically using more than 20 radar images. The calculation formula is as follows:

(1) $$D_{A}=\frac{\sigma_{A}}{m_{A}}.$$

where $\sigma_{A}$ represents the standard deviation of the amplitude time series of the target point, and $m_{A}$ represents the mean of the amplitude time series of the target point. By setting an amplitude deviation threshold $D_{threshold}$, points with $D_{A}<D_{threshold}$ are considered candidate PS points. In this study, the threshold was set to 0.25.

The coherence coefficient method estimates the coherence coefficient of a pixel using the information from neighboring pixels. The formula is as follows:

(2) $$r=\frac{\left|\Sigma_{i=1}^{m}\Sigma_{j=1}^{n}M\left(i,j\right)S^{*}\left(i,j\right)\right|}{\sqrt{\sum_{i=1}^{m}\;\sum_{j=1}^{n}\;\left|M\left(i,j\right){|}^{2}\Sigma_{i=1}^{m}\Sigma_{j=1}^{n}\right|S\left(i,j\right){|}^{2}}}.$$

where M and S are the local pixel information of the radar’s master and slave images, respectively, and * denotes complex conjugate multiplication. m×n represents the size of the moving window [20]. In this study, points with a coherence coefficient greater than 0.9 were selected as candidate PS points. Figure 3b shows the selected amplitude deviation map, and Figure 3c presents the selected coherence coefficient map. Using the aforementioned dual-threshold method, a total of 128,276 PS points were selected. As shown in Figure 3d, most points in the study area were selected.

## 3. Method

### 3.1. Signal Model

The phase obtained by differential interferometry from two radar images acquired at different times over the same location can be modeled as:

(3) $$\begin{array}{ll} \Delta \varphi \left(\vec{r},t\right) & =\Delta \varphi_{sca}+\frac{4\pi f_{c}}{c}\Delta r+\Delta \varphi_{atm}\left(\vec{r},t\right)+\Delta \varphi_{noi}\\ & =\Delta \varphi_{sca}+\Delta \varphi_{defo}+\Delta \varphi_{atm}+\Delta \varphi_{noi}. \end{array}$$

where $\Delta \varphi_{sca}$ is the backscattering phase component, $\Delta \varphi_{defo}$ is the deformation phase component, $\Delta \varphi_{atm}$ is the atmospheric phase delay component, and $\Delta \varphi_{noi}$ is the thermal noise phase component [21].

The atmospheric delay phase can be obtained by integrating the refractive index n along the propagation path L.

(4) $$\varphi_{atm}\left(\vec{r},t\right)=\frac{4\pi f_{c}}{c}\int_{L}n\left(\vec{r},t\right)dl.$$

Thus, $\Delta \varphi_{atm}$ can be derived as:

(5) $$\Delta \varphi_{atm}=\frac{4\pi f_{c}}{c}\int_{L}\Delta n\left(\vec{r},t\right)dl.$$

where Δn represents the change in the refractive index. For ease of calculation, the refractive index n is generally expressed as the refractivity N, with the relationship:

(6) $$N=\left(n-1\right)\times 10^{6}.$$

Therefore, we have:

(7) $$\Delta \varphi_{atm}=10^{-6}\times \frac{4\pi r_{s}}{\lambda }\int_{L}\Delta N\left(\vec{r},t\right)dl.$$

According to the International Telecommunication Union, the relationship between the air refractivity N and meteorological parameters can be approximated as follows:

(8) $$N=1+10^{-6}\cdot \left(77.6\frac{P}{T}-5.6\frac{e}{T}+3.75\times 10^{5}\frac{e}{T^{2}}\right).$$

where P and e represent the dry air pressure (in hectopascals, hPa) and wet air pressure (in hectopascals, hPa), respectively, and T is the absolute temperature (in Kelvin, K). Parameter e can be calculated using T, P, and relative humidity (RH%).

Under general weather conditions, when conducting short-term monitoring over small areas, the atmosphere within the region can be considered a homogeneous medium. The refractivity N does not change with the monitoring distance r, leading to the simplified function of time only:

(9) $$\varphi \left(t\right)=\frac{4\pi r_{s}}{\lambda }N\left(t\right).$$

Therefore, in traditional model–based methods, models related to distance have been established [12,22]:

(10) $$\Delta \varphi_{atm}=\beta_{1}\cdot r.$$

(11) $$\Delta \varphi_{atm}=\beta_{1}\cdot r+\beta_{2}\cdot r^{2}.$$

Segmented linear model [23]:

(12) $$\begin{array}{cc} y_{1} & =a_{1}r+c_{1},r<w\\ y_{2} & =a_{2}r+c_{2},r\geq w. \end{array}$$

Height-related model [13]:

(13) $$\Delta \varphi_{atm}=\beta_{1}\cdot r+\beta_{2}\cdot r\cdot \Delta h.$$

Angle-related model [24,25]:

(14) $$\Delta \varphi_{atm}=\beta_{0}+\beta_{1}\cdot r+\beta_{2}\cdot r\cdot \sin\theta .$$

Taking the quadratic distance model as an example to fit the atmospheric phase screen, a sufficient number of PS points is needed to construct the linear equation system:

(15) ΔΦ=Xβ+ε.

where:

(16) $$\left.\left.\Delta \Phi =\left[\begin{array}{c} \Delta \varphi_{1}\\ \Delta \varphi_{2}\\ \vdots \\ \Delta \varphi_{m} \end{array}\right.\right],X=\left[\begin{array}{ccc} 1 & r_{1} & r_{1}^{2}\\ 1 & r_{2} & r_{2}^{2}\\ \vdots & \vdots & \vdots \\ 1 & r_{m} & r_{m}^{2} \end{array}\right.\right]\left.,\beta =\left[\begin{array}{c} \beta_{0}\\ \beta_{1}\\ \beta_{2} \end{array}\right.\right],\left.\varepsilon =\left[\begin{array}{c} \varepsilon_{1}\\ \varepsilon_{2}\\ \vdots \\ \varepsilon_{m} \end{array}\right.\right].$$

where m is the number of PS points and ${\Delta \Phi }_{i}$ and $r_{i}$ are the phase and distance of the i-th PS point relative to the radar, respectively. **X** is an m×3 observation matrix, β is a 3×1 vector to be estimated, and ε is an m×1 vector containing random errors. The unknown vector β can be estimated using least squares regression:

(17) $$\hat{\beta }={\left(X^{T}X\right)}^{-1}X^{T}\Delta \Phi .$$

The estimated atmospheric phase is:

(18) $$\Delta {\hat{\Phi }}_{AP}=X\hat{\beta .}$$

By subtracting the estimated atmospheric phase from the interferometric phase, the compensated phase is obtained. The unbiased estimate of variance is given by:

(19) $$S^{2}=\frac{\Delta \Phi^{T}\Delta \Phi -{\hat{\beta }}^{T}X^{T}\Delta \Phi }{m-3}.$$

After initial compensation, some unreliable PS points, such as those in deformation areas or heavily noise-affected points, may remain. To ensure the accuracy of the estimation results, PS points that do not meet the condition in Equation (20) are removed, and a second estimation is performed using the remaining reliable PS points [14]:

(20) $$\left|\Delta \Phi -\Delta {\hat{\Phi }}_{AP}\right|<2\cdot S.$$

### 3.2. Traditional Method

**Figure 4:**
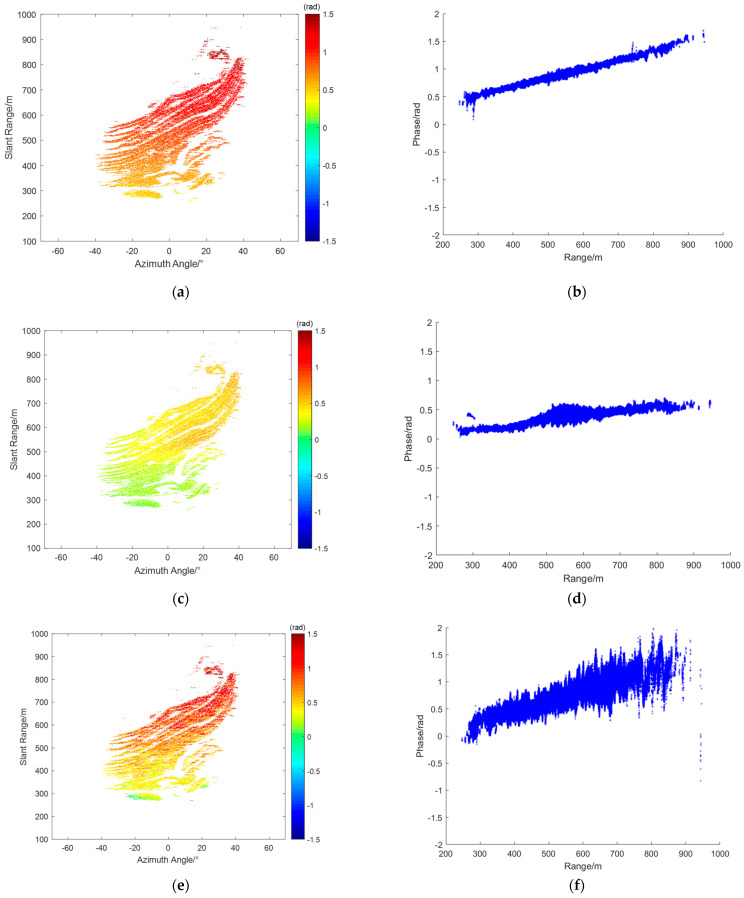

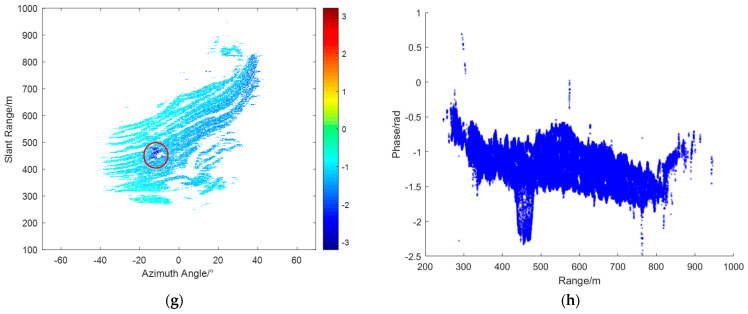
Interferograms: (**a**) ${IM}^{\left(a\right)}$, (**c**) ${IM}^{\left(b\right)}$, (**e**) ${IM}^{\left(c\right)},\;$and (**g**) ${IM}^{\left(d\right)}$; SD over range: (**b**) ${SD}^{\left(a\right)}$, (**d**) ${SD}^{\left(b\right)}$, (**f**) ${SD}^{\left(c\right)},\;$and (**h**) ${SD}^{\left(d\right)}$.

From the radar data collected during this experiment, interferograms were obtained, and four representative interferograms were selected for atmospheric phase correction (APC) processing. Figure 4a,c,e,g represent the interferograms ${IM}^{\left(a\right)}$, ${IM}^{\left(b\right)}$, ${IM}^{\left(c\right)},\;$and ${IM}^{\left(d\right)}$, respectively. Figure 4b,d,f,h show the corresponding range scatter plots ${SD}^{\left(a\right)},{SD}^{\left(b\right)},{SD}^{\left(c\right)},\;{and\;SD}^{\left(d\right)}$. As shown, the unfolded phases of all PS points fell between −2.5 radians and 2 radians.

From the interferogram ${IM}^{\left(a\right)}$ and its scatter plot ${SD}^{\left(a\right)}$, it can be observed that the phase increases linearly along the range axis. In this case, the assumption of atmospheric homogeneity holds well. This means that atmospheric correction techniques based on linear models can be effectively applied. In the interferogram ${IM}^{\left(b\right)}$, the atmospheric phase variation is not purely linear. The scatter plot ${SD}^{\left(b\right)}$ shows a significant curved trend in the range direction. Additionally, the interferogram reveals noticeable azimuthal atmospheric components. This complexity indicates that the atmospheric phase variation is influenced by multiple factors, making it necessary to apply more sophisticated models that can account for these non–linear variations. From interferogram ${IM}^{\left(c\right)}$, it can be seen that it can be divided at around 500 m. The region with a distance greater than 500 m experiences more severe atmospheric effects. Additionally, from the corresponding scatter plot ${SD}^{\left(c\right)}$, it can be observed that the atmospheric phase varies linearly with the distance. In interferogram ${IM}^{\left(d\right)}$, the red circle marks the location of a landslide that occurred during the observation period. The phase at this location differs significantly from corresponding locations in other interferograms. The scatter plot ${SD}^{\left(d\right)}$ shows more complex phase variations compared to ${IM}^{\left(a\right)}$, ${IM}^{\left(b\right)},\;$and ${IM}^{\left(c\right)}$. The intricate phase changes observed here are likely due to both the deformation caused by the landslide and the varying atmospheric conditions. Based on the specific characteristics of each interferogram, different models are used for APC.

**Figure 5:**
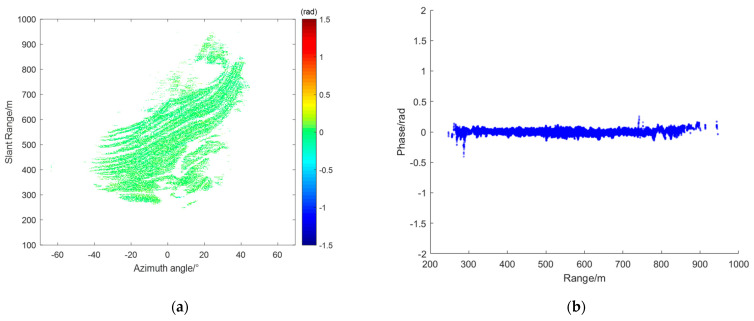
Atmospheric phase compensation (APC) results of ${IM}^{\left(a\right)}$: (**a**)$\;{CIM}^{\left(a\right)}$; (**b**)$\;{CSD}^{\left(a\right)}$.

For interferogram ${IM}^{\left(a\right)}$, where the phase increases linearly along the range axis, the second-order range model is used for compensation. Figure 5a shows the compensated interferogram ${CIM}^{\left(a\right)}$, and Figure 5b shows the corresponding scatter plot ${CSD}^{\left(a\right)}$. As shown, the phases of all PS points are close to 0 rad, indicating that the linear phase along the range axis was well compensated.

**Figure 6:**
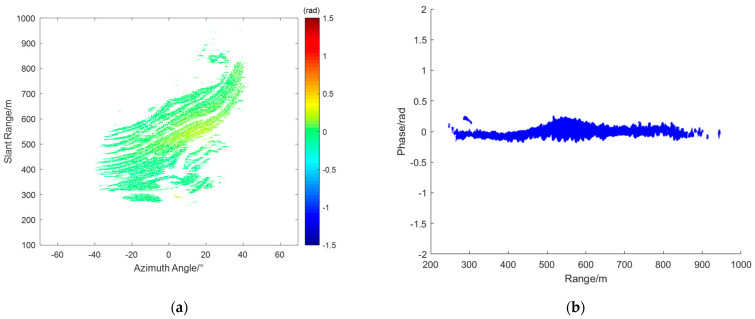
Atmospheric phase compensation (APC) results of ${IM}^{\left(b\right)}$: (**a**) ${CIM}^{\left(b\right)}$; (**b**) ${CSD}^{\left(b\right)}$.

The range & azimuth model is used to compensate for the atmospheric phase in interferogram ${IM}^{\left(b\right)}$. Figure 6a shows the compensated interferogram ${CIM}^{\left(b\right)}$, and Figure 6b shows the corresponding scatter plot ${CSD}^{\left(b\right)}$. It can be seen that the phases of all PS points are between −0.5 rad and 0.5 rad, indicating good compensation.

**Figure 7:**
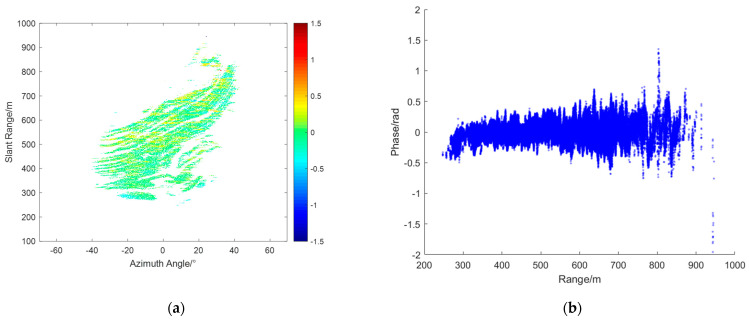
Atmospheric phase compensation (APC) results of ${IM}^{\left(c\right)}$: (**a**) ${CIM}^{\left(c\right)}$; (**b**) ${CSD}^{\left(c\right)}$.

For interferogram ${IM}^{\left(c\right)}$, its characteristics allow for atmospheric phase correction using a piecewise linear model. In this paper, the piecewise distance is set to 550 m. Figure 7a shows the compensated interferogram ${CIM}^{\left(c\right)}$, and Figure 7b shows the corresponding scatter plot ${CSD}^{\left(c\right)}$. As can be seen, most of the PS points have phases between −0.5 and 0.5 radians, with some noise points present, but overall, good compensation was achieved.

**Figure 8:**
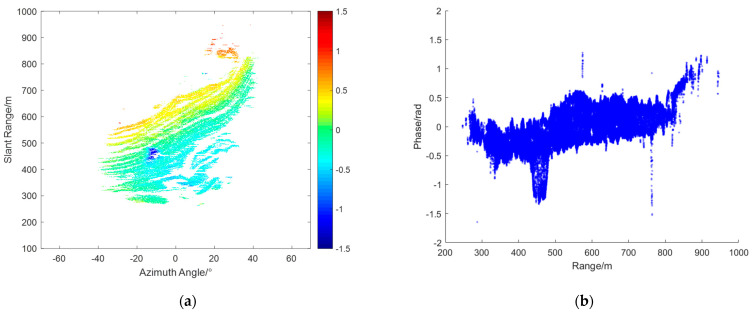
Atmospheric phase compensation (APC) results of ${IM}^{\left(d\right)}$: (**a**) ${CIM}^{\left(d\right)}$; (**b**) ${CSD}^{\left(d\right)}$.

For interferogram ${IM}^{\left(d\right)}$, because of the significant azimuthal component, the range&azimuth model is applied for atmospheric phase correction. Figure 8a shows the compensated interferogram ${CIM}^{\left(d\right)}$, and Figure 8b shows the corresponding scatter plot ${CSD}^{\left(d\right)}$. As can be seen, most of the PS points have phases between −1.5 and 1.5 radians, indicating that the atmospheric phase was been well compensated. This result suggests that traditional model–based methods are not suitable for handling complex interferograms effectively.

### 3.3. Improved Method

Traditional atmospheric phase removal techniques often employ global correction methods that are effective under relatively stable and homogeneous atmospheric conditions. However, these methods fail to account for the impact of terrain complexity and atmospheric variability on phase errors, particularly in areas with significant topographical relief or rapidly changing atmospheric conditions. The method proposed in this section calculates the spatial autocorrelation of each sub–block based on uniform partitioning and further divides each sub–block into multiple regions using the region growing algorithm. Then, by comparing the phase averages of different regions in adjacent sub-blocks, merging is performed to complete the clustering process. For each cluster, regression fitting using the range & azimuth model is conducted to obtain the atmospheric phase screen of the stable regions, followed by IDWI interpolation to obtain a complete atmospheric phase screen. This approach allows for a more detailed analysis of local characteristics in interferograms, capturing the features of atmospheric phase errors more precisely.

#### 3.3.1. Local Atmospheric Phase Feature Analysis and Region-Growing Classification

To analyze the local characteristics of the atmospheric phase in detail, the interferogram is first uniformly divided into multiple grids, with the size of each grid determined based on the actual data image size. After division, spatial autocorrelation analysis is performed within each grid. Spatial autocorrelation analysis is a statistical method that measures the phase correlation between pixels in an interferogram by calculating the spatial autocorrelation function (ACF). This method quantifies the correlation of atmospheric phase errors at different spatial scales—in other words, the local phase consistency within each grid of the interferogram. Specifically, for a given pixel in each grid, the ACF calculation analyzes its phase relationship with the surrounding pixels. A high ACF value indicates a high degree of phase consistency within the grid, while a low ACF value indicates significant phase variations.

The ACF formula is expressed as:

(21) $$ACF\left(x,y\right)=\sum_{dx=-L}^{L}\;\sum_{dy=-L}^{L}\;\varphi \left(x,y\right)\cdot \varphi \left(x+dx,y+dy\right).$$

where $ACF\left(x,y\right)$ represents the autocorrelation value at coordinates $\left(x,y\right)$, and $\varphi \left(x,y\right)$ represents the phase value at coordinates $\left(x,y\right)$. dx and dy represent displacements in the x and y directions, respectively, ranging from −L to L, with L being the maximum displacement range considered for phase correlation calculation. After calculating the ACF value for each element in the grid, normalization is performed using Equation (22) to ensure the comparability of autocorrelation values between different grids, with the normalized range being [−1,1].

(22) $$ACF_{nor}\left(x,y\right)=\frac{ACF\left(x,y\right)}{max(ACF(x,y){)}_{x,y}}.$$

**Figure 9:**
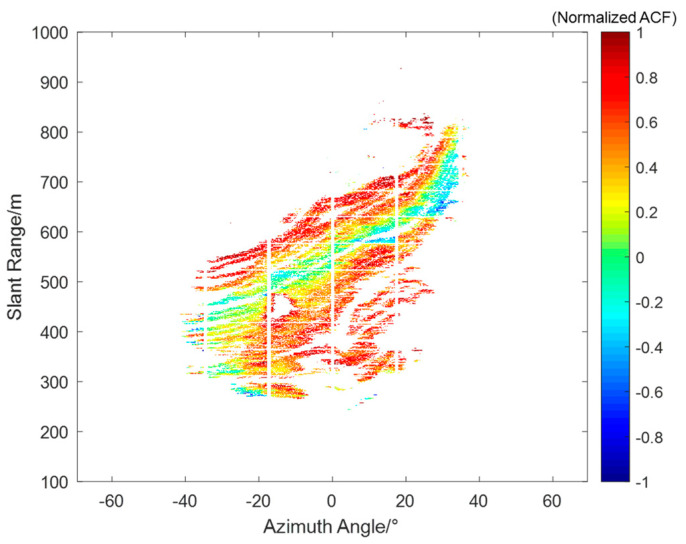
Results of uniform partitioning and spatial autocorrelation.

Based on the ACF results, phase variation patterns and trends within the grid can be identified, distinguishing between uniform regions and regions with significant phase variations. For example, interferogram ${IM}^{\left(c\right)}$ is initially segmented, and spatial autocorrelation is calculated for each grid, as shown in Figure 9. Each grid corresponds to a calculated autocorrelation value, with colors indicating the phase variation trend within each grid.

Next, region growing algorithms are applied to classify the ACF-processed results, identifying and marking regions with similar phase characteristics [26]. Within each sub-block, local maxima of the spatial autocorrelation values are first used as seed points. Starting from these seed points, the eight neighboring pixels are examined to see whether they meet the following condition and are added to the region containing the current seed point:

(23) $$\left|{ACF}_{current}-{ACF}_{max}\right|\leq \alpha \times \left|{ACF}_{seed}\right|.$$

where ${ACF}_{current}$ represents the current pixel’s autocorrelation value; ${ACF}_{max}$ represents the maximum value within the current sub–block; ${ACF}_{seed}$ is the seed point’s autocorrelation value; and α is an empirically determined similarity threshold ratio based on previous observations and experimental data, with a range from 0 to 1. The threshold is used to adjust the sensitivity of the region growing algorithm and control the number and size of generated regions, and it is an empirical threshold. Specifically, a smaller α value increases the algorithm’s sensitivity to phase changes, triggering new region growth even for minor phase differences, resulting in more segmented regions. In this α value scenario, since the subsequent region merging involves combining regions between adjacent grids, having too many subdivisions within a grid can lead to many regions being unmerged, thus affecting the final clustering effect. Conversely, a larger α value reduces the algorithm’s sensitivity to phase changes, resulting in fewer segmented regions. For grids with significant phase changes, this situation fails to accurately describe the variation patterns, weakening the fitting effect of the atmospheric phase screen. An appropriate α value allows grids with small phase changes and stable ACF values to maintain larger regions. Conversely, for grids with significant phase changes and fluctuating ACF values, the algorithm can more easily identify different phase patterns, generating more segmented regions to accurately describe these changes. In our experiments, based on previous research and experimental data, we selected α as 0.4. By adjusting the α value, we observed that when α is less than 0.3, some segmented regions are too numerous, making effective subsequent processing difficult; when α is greater than 0.5, some segmented regions are too few, failing to accurately capture phase changes. Therefore, 0.4 is a balance point that effectively captures phase changes without leading to excessive segmentation.

After the above processing, some regions may have too few pixels to be useful for subsequent data processing and analysis. To ensure the validity of the data and the accuracy of the analysis, these regions (referred to as “small regions”) need to be merged. First, a pixel count threshold is set, which in this paper is 10% of the current region’s valid pixel count, used to define which regions are considered small regions. These small regions are then merged into the nearest region within the sub–block for subsequent fitting processing.

**Figure 10:**
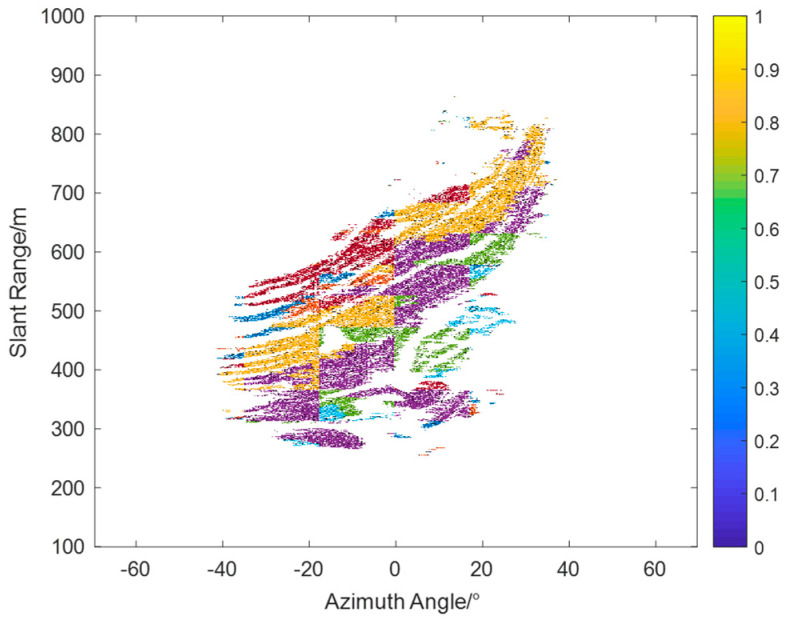
Spatial autocorrelation clustering results.

Furthermore, the average phase of each region identified by the region growing algorithm is calculated, and the average phases of different regions in adjacent sub–blocks are compared. Regions meeting the following condition are merged across sub-blocks:

(24) $$\left|\varphi_{meanA}-\varphi_{meanB}\right|<\lambda \cdot \max\left(\left|\varphi_{meanA}\right|,\left|\varphi_{meanB}\right|\right).$$

where $\varphi_{meanA}\;$is the phase average of the first block, and $\varphi_{meanB}$ is the phase average of the second adjacent block. λ is an empirical threshold for controlling the merging of regions between adjacent grids, ranging from 0 to 1. A smaller λ value means that only regions with very similar means will be merged, which may result in many regions being excluded from merging and thus affecting the clustering effect. Conversely, a larger λ value may lead to excessive merging, causing some important details to be lost. Therefore, this paper chooses 0.6. Through experiments, we found that when λ is less than 0.5, the number of merged regions is too high, resulting in unstable analysis results; when λ is greater than 0.7, excessive merging occurs, and many details are lost. Therefore, we chose 0.6 as the value of λ to ensure that the number of regions is effectively reduced without losing important details. After merging, regions with similar phase trends and phase values are combined to complete the clustering. Figure 10 shows the clustering results, with different colors distinguishing adjacent subclasses.

#### 3.3.2. Atmospheric Phase Estimation and Correction

After region merging, multivariate regression fitting is performed for each sub–class according to the following model:

(25) $$\phi =\beta_{0}+\beta_{1}r+\beta_{2}\sin\left(\theta \right).$$

After initial atmospheric phase removal using the second-order range model, unstable points in deformation areas or those heavily affected by noise are removed. To obtain a complete atmospheric phase screen, inverse distance weighting interpolation is applied after multivariate regression fitting. Inverse distance weighting interpolation defines weights based on the distance between known points and the interpolation point, using the surrounding known points to calculate the interpolated phase value. The closer the distance, the greater the weight. The formula is:

(26) $$w_{i}=\frac{1}{d_{i}^{p}}\;.$$

where $w_{i}$ is the weight of the i-th sample point; $d_{i}$ is the distance between the sample point and the point to be interpolated; and p is an adjustable parameter, typically set to 2 (Euclidean distance). After calculating the weight of each known sample point, the phase values are weighted and averaged to obtain the phase value of the interpolated point:

(27) $$z\left(x,y\right)=\frac{\sum_{i=1}^{N}\;w_{i}z_{i}}{\sum_{i=1}^{N}\;w_{i}}.$$

where N is the number of known sample points, $\left(x_{i},y_{i}\right)$ are the coordinates of the i-th sample point, $w_{i}$ is the weight, and $z_{i}$ is the function value. The phase value of the interpolated position is z(x,y). After interpolation, a complete estimated atmospheric phase screen is obtained. By subtracting this atmospheric phase screen from the interferometric phase, the compensation is completed.

**Figure 11:**
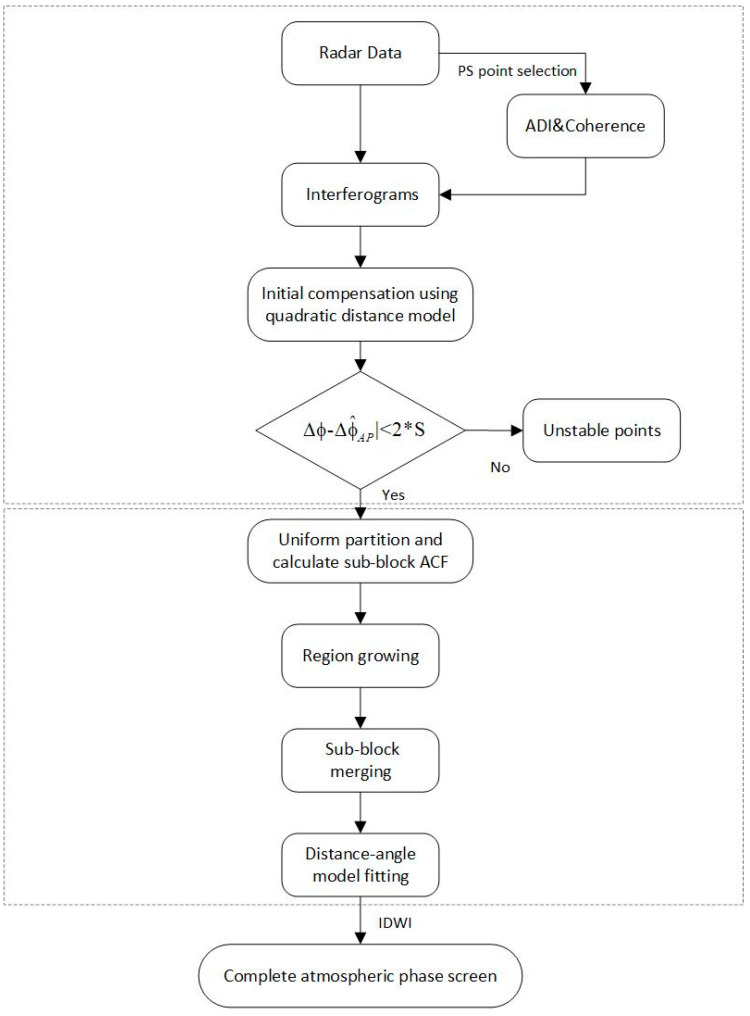
Flowchart of the proposed method in this paper.

Therefore, the method proposed in this paper consists of two steps. First, the interferogram is initially compensated using the second-order range model, and PS points that do not meet the condition in Equation (20) are removed. Then, secondary compensation is performed using the spatial autocorrelation clustering method. The detailed procedure is illustrated in Figure 11, which provides a clear visual overview of the two-step process.

## 4. Experimental Results

To thoroughly evaluate the effectiveness and superiority of the spatial autocorrelation clustering method proposed in this paper for removing atmospheric phase errors, this section processes interferograms ${IM}^{\left(a\right)}$, ${IM}^{\left(b\right)}$, ${IM}^{\left(c\right)},\;$and ${IM}^{\left(d\right)}\;$using the proposed method. The results are then compared, both quantitatively and qualitatively, with those obtained using the second-order range model, the range&azimuth model, and the piecewise linear model.

**Figure 12:**
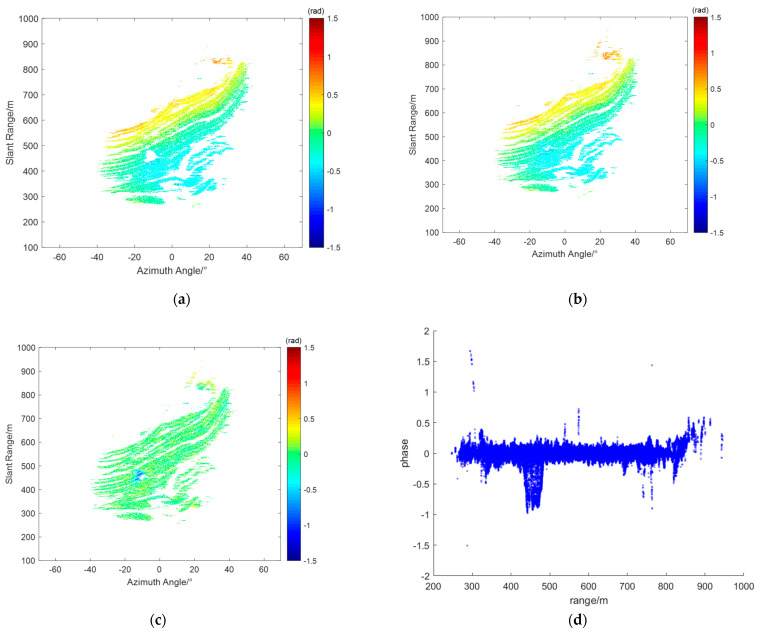
Analysis results of the interferogram ${IM}^{\left(d\right)}$: (**a**) preliminary fitting results; (**b**) complete estimation of the atmospheric phase screen; (**c**) compensation results of the proposed method; (**d**) corresponding compensated SDs.

Taking the interferogram ${IM}^{\left(d\right)}$, which cannot be compensated by traditional methods, as an example, we first use all selected PS points for initial estimation, retaining reliable PS points for a second estimation. Figure 12a shows the initial estimation results, where unstable points at the landslide location and some noisy points have been removed. Figure 12b shows the complete atmospheric phase screen obtained after the second estimation. Figure 12c shows the interferogram after compensation using the proposed method, and Figure 12d shows the corresponding scatter plot. It can be seen that most PS point phases, except those in the landslide area, are between −0.5 and 0.5 rad, indicating that the atmospheric phase component has been well compensated. A few PS points deviate from 0 rad, which may be due to noise.

For interferograms ${IM}^{\left(a\right)}$,${IM}^{\left(b\right)},\;$and ${IM}^{\left(c\right)}$, which can be well compensated by the second-order range model, the range&azimuth model, and the piecewise linear model, the proposed method can also provide better compensation. Table 2 shows the root mean square error (RMSE) after compensation for interferograms ${IM}^{\left(a\right)}$, ${IM}^{\left(b\right)}$, ${IM}^{\left(c\right)}$, and ${IM}^{\left(d\right)}$ using the four methods. The smaller the value, the better the compensation performance. Model 1 is the second-order range model, Model 2 is the range&azimuth model, Model 3 is the piecewise linear model, and Model 4 is the method proposed in this paper. It can be seen that the proposed method yields a root mean square error (RMSE) that is consistently lower than both the second–order range model and the range&azimuth model. For the interferogram ${IM}^{\left(d\right)}$, which cannot be effectively handled by the three traditional models, the proposed method achieves an RMSE of 0.0329, significantly lower than the results from the traditional models. This demonstrates the effectiveness of the proposed method in compensating for complex atmospheric phase errors.

**Figure 13:**
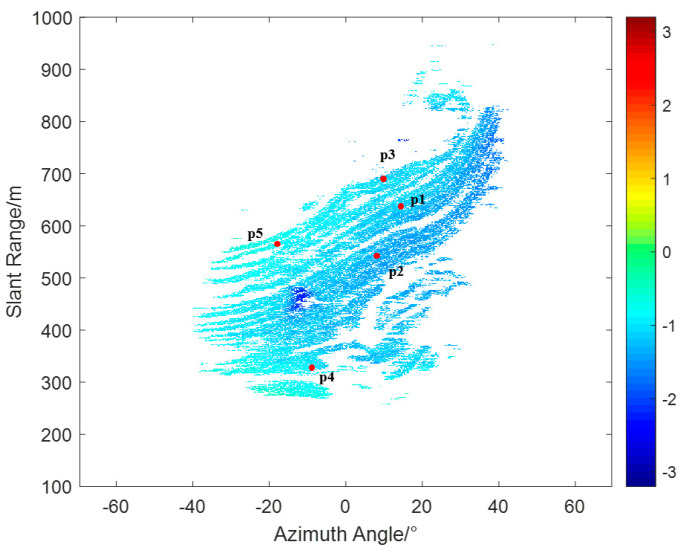
Chosen stable PS.

To further validate the effectiveness of the proposed method, five PS points (p1–p5) were selected from different locations on the slope interferogram, as shown by the red dots in Figure 13. These PS points have amplitude deviation indices of less than 0.05 and coherence coefficients greater than 0.95, indicating that they are stable points whose phase changes are primarily caused by atmospheric variations. The 224 interferograms obtained from the experiment were processed using both traditional methods and the proposed method.

**Figure 14:**
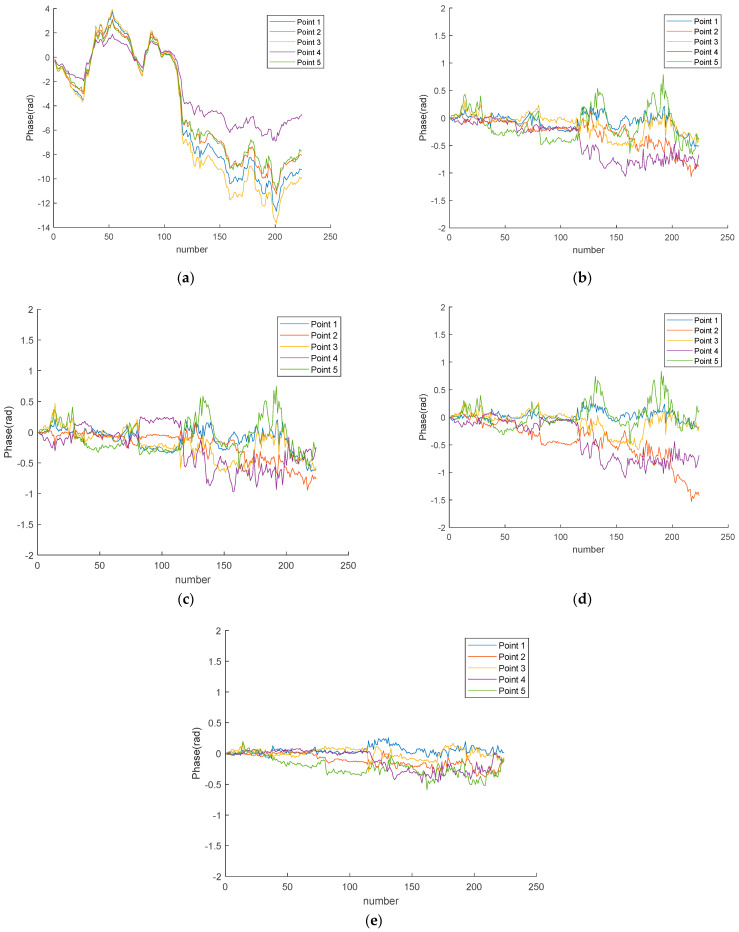
P1–P5 phase curve: (**a**) The original phase curve. (**b**) The phase curve after the quadratic distance model compensation. (**c**) The phase curve after the distance angle model compensation. (**d**) The phase curve after the piecewise linear model compensation. (**e**) The phase curve compensated by this method.

Figure 14a shows the phase accumulation curves for P1–P5 without any compensation. It can be seen that these five points exhibit similar phase variation trends, with values reaching up to 14 radians. For Ku–band radar, this corresponds to an error range of 18.48 mm to 27.86 mm. In the later stages of the observation experiment, the atmospheric variations at these five points are more severe compared to the initial stages of the observation. Figure 14b shows the phase variation curves after compensation using the second-order range model, Figure 14c shows the phase variation curves after compensation using the range&azimuth model, Figure 14d shows the phase variation curves after compensation using the piecewise linear model, and Figure 14e shows the phase variation curves after compensation using the proposed method. It is evident that, after compensation using the proposed method, the phases of the stable points are close to 0 radians, with minimal fluctuation.

**Figure 15:**
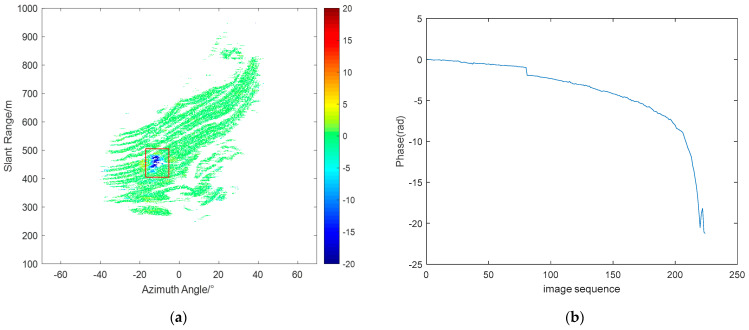
(**a**) Phase accumulation in landslide area. (**b**) Phase accumulation curve.

Figure 15a shows the phase accumulation map processed using the proposed method, with the red rectangle indicating the landslide location, where blue represents the direction away from the radar. Figure 15b presents the phase accumulation curve at the landslide location. It can be seen that the maximum cumulative deformation during the experiment reached approximately 42 mm. On–site workers have confirmed and identified the occurrence of the landslide.

## 5. Discussion

When using ground–based synthetic aperture radar (GB-SAR) to monitor the study area, the spatiotemporal variations in atmospheric conditions caused delays in the propagation of radar electromagnetic waves, resulting in atmospheric phase errors. Generally, the atmosphere can be assumed to be homogeneous and corrected using models based on distance or height. However, rapid weather changes or steep observation areas can lead to complex atmospheric phases that do not exhibit simple variations along distance, azimuth, or height in the interferogram. Under such conditions, the assumption of atmospheric homogeneity becomes invalid. Over time, these complex atmospheric effects can severely impact the monitoring of deformation areas, leading to misinterpretations.

To address these complex atmospheric phases, we propose a method based on spatial autocorrelation. This method divides the interferogram into equally sized grids and calculates the spatial autocorrelation for each grid, as shown in Figure 9. This allows for the analysis of phase variation trends within each grid. Based on the corresponding phase variations, the grids are further classified, and neighboring grid subclasses are merged through averaging to complete the clustering, as shown in Figure 10. After clustering, each class is fitted using the range&azimuth regression model, and inverse distance weighting interpolation (IDWI) is used to obtain a complete atmospheric phase screen. The advantage of this method lies in its ability to analyze phase consistency in local areas and handle atmospheric phases under complex atmospheric conditions.

In the experiment, we compared the traditional model–based method with the proposed method. Table 2 shows the root mean square error (RMSE) values after compensation for each method, demonstrating that the proposed method achieved the smallest RMSE. Additionally, five stable points were selected to calculate their phase accumulation curves, with most phase variations attributed to atmospheric influences. As shown in Figure 14a, the atmospheric phase variations during the experiment were significant. After compensating using the model–based methods, the phase curve variations remained large. Figure 14d shows the phase accumulation curves after compensation using the proposed method, clearly demonstrating the effectiveness of this method.

## 6. Conclusions

This paper proposes a spatial autocorrelation clustering method for removing atmospheric phase (AP) errors in ground–based synthetic aperture radar (GB-SAR). Observations of open–pit mines using ground–based radar revealed that AP might exhibit complex spatiotemporal variations due to rapid weather changes or steep terrain. Traditional model–based methods, such as the second–order range model, the range&azimuth model, and the piecewise linear model, cannot accurately fit this complex atmosphere.

The proposed method clusters grids based on ACF after uniformly dividing the interferogram into blocks. In the experiment, four representative interferograms were processed for their atmospheric components, and the analysis of the compensation results demonstrated that the proposed method can achieve better compensation effects. Five high–quality permanent scatterer (PS) points were selected for quantitative analysis, and the experimental results showed that the method effectively removed complex atmospheric phases in radar images. Compared to traditional global phase removal methods, the proposed method significantly improved the accuracy and reliability of phase correction through local analysis and processing.

## Figures and Tables

**Table 1:** System parameters.

Parameter	Value
Range Resolution	0.2 m
Azimuth Resolution	0.3°
Band	Ku
Sampling Interval	20 min

**Table 2:** The root mean square error of the phase diagram is mitigated through various methods.

	Model 1	Model 2	Model 3	Model 4
${IM}^{\left(a\right)}$	0.0103	0.0108	0.0085	0.0077
${IM}^{\left(b\right)}$	0.0656	0.0653	0.0407	0.0352
${IM}^{\left(c\right)}$	0.0479	0.0454	0.0439	0.0376
${IM}^{\left(d\right)}$	0.0738	0.0732	0.0759	0.0329

## Data Availability

Data are contained within the article.
